# Supplementary material for: Biocatalytic Potential of Native Basidiomycetes from Colombia for Flavour/Aroma Production
Source: Molecules. 2020 Sep 22;25(18):4344. doi: 10.3390/molecules25184344 (PMC7570902; doi:10.3390/molecules25184344)
Supplement: Supplementary file 1 [file molecules-25-04344-s001.pdf]

molecules

Article

Biocatalytic potential of native Basidiomycetes from Colombia for flavor/aroma production

David A. Jaramillo 1, María J. Méndez 1, Aída-M. Vasco-Palacios 2, Gabriela Vargas1, Andrés Ceballos 1, Elena E. Stashenko 3, and Nelson H. Caicedo 1,*

[1] Department of Biochemical Engineering, Universidad Icesi, Calle 18 No. 122-135 Pance, Cali, Colombia; nhcaicedo@icesi.edu.co
[2] Grupo de Microbiología Ambiental - BioMicro, Escuela de Microbiología, Universidad de Antioquia, UdeA, Calle 70 No. 52-21, Medellín, Colombia ; aida.vasco@udea.edu.co
[3] Universidad Industrial de Santander. Chromatography and Mass Spectrometry Center; elena@tucan.uis.edu.co
* Correspondence: nhcaicedo@icesi.edu.co

Supplementary Materials: The following are available online at www.mdpi.com/1420-3049/25/18/4344/s1

Figure S1a to S1h: Compound identification of the eight peaks shown in figure 6 using headspace gas chromatography-mass spectrometry (GC-MS) technique. **Figure S1a:** Peak 1 (7.44 min), **Figure S1b:** Peak 2 (10.13 min), **Figure S1c:** Peak 3 (10.78 min), **Figure S1d:** Peak 4 (11.54 min), **Figure S1e:** Peak 5 (Average of 20.323 to 20.36 mi), **Figure S1f:** Peak 6 (21.344 min), **Figure S1g:** Peak 7 (21.35 min), **Figure S1h:** Peak 8 (26.305 min).

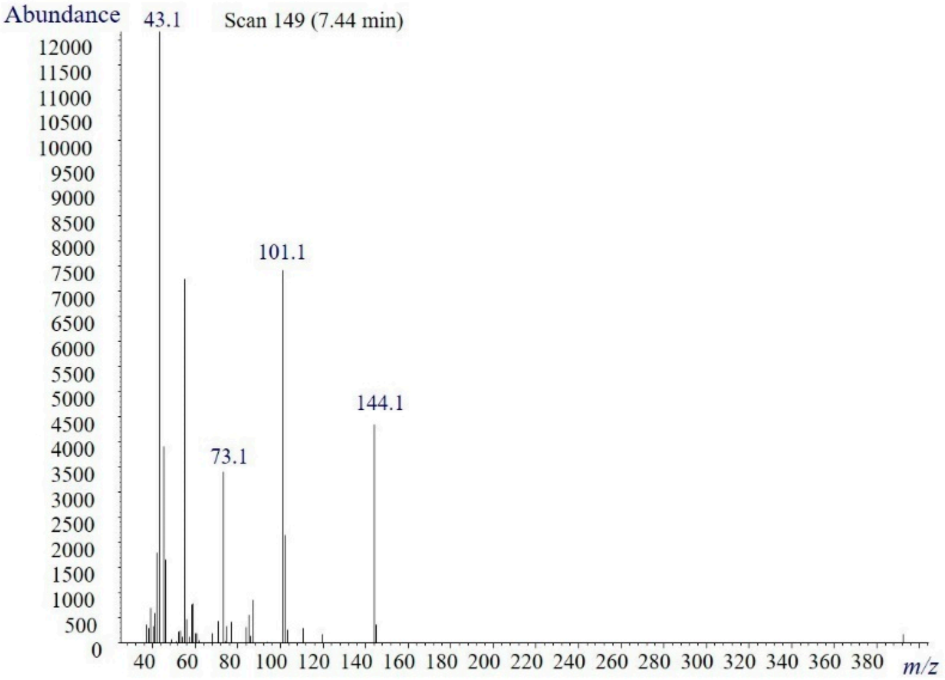

Figure S1a. Mass spectrum of the peak N° 1 (7.44 min) shown in figure 6 by using gas chromatography-mass spectrometry (GC-MS) technique.

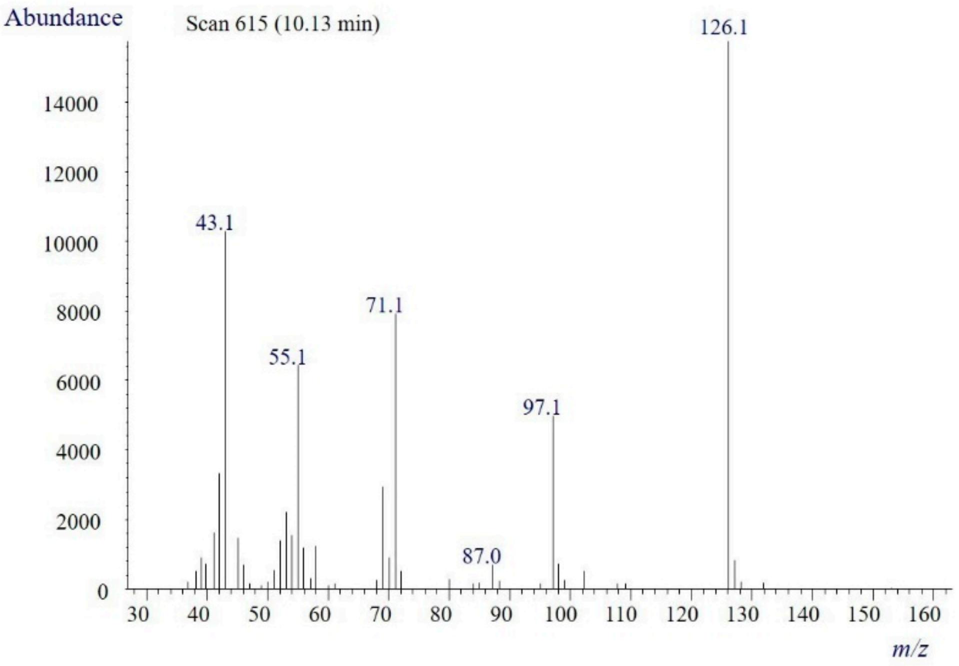

Figure S1b. Mass spectrum of the peak N° 2 (10.13 min) shown in figure 6 by using gas chromatography-mass spectrometry (GC-MS) technique.

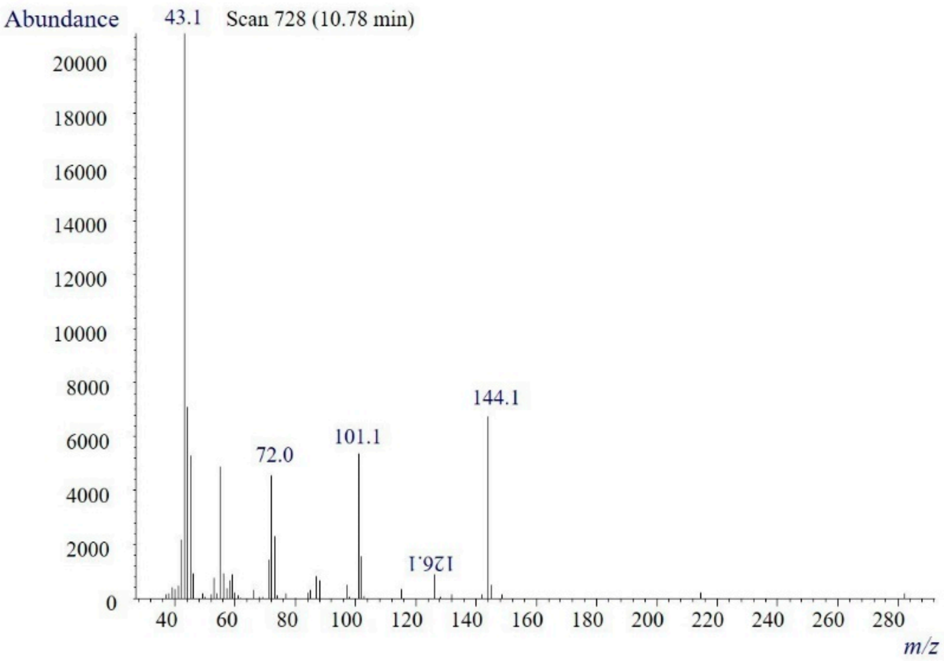

Figure S1c. Mass spectra of the peak N° 3 (10.78 min) shown in figure 6 using headspace gas chromatography-mass spectrometry (GC-MS) technique.

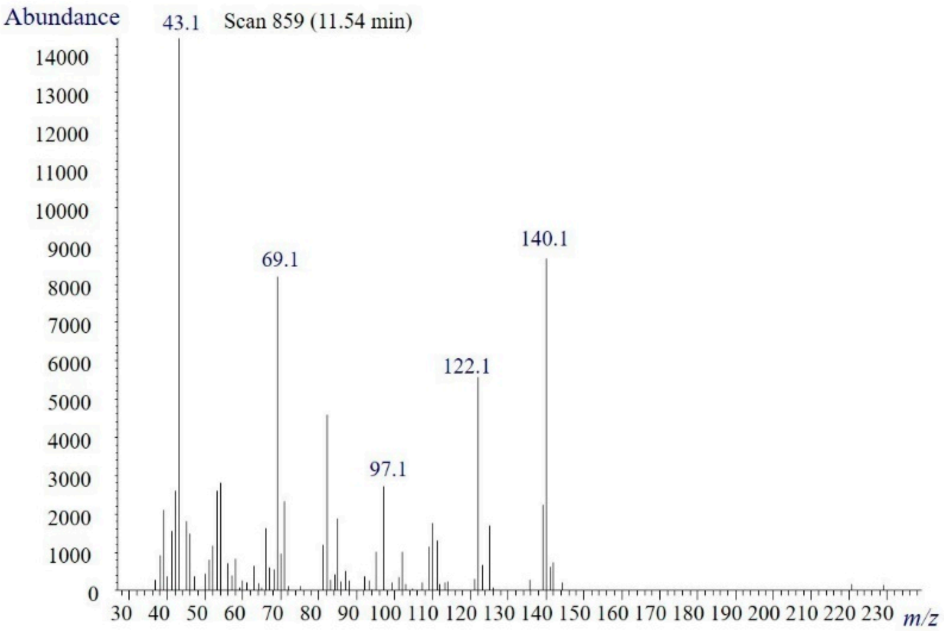

Figure S1d. Mass spectrum of the peak N° 4 (11.54 min) shown in figure 6 by using gas chromatography-mass spectrometry (GC-MS) technique.

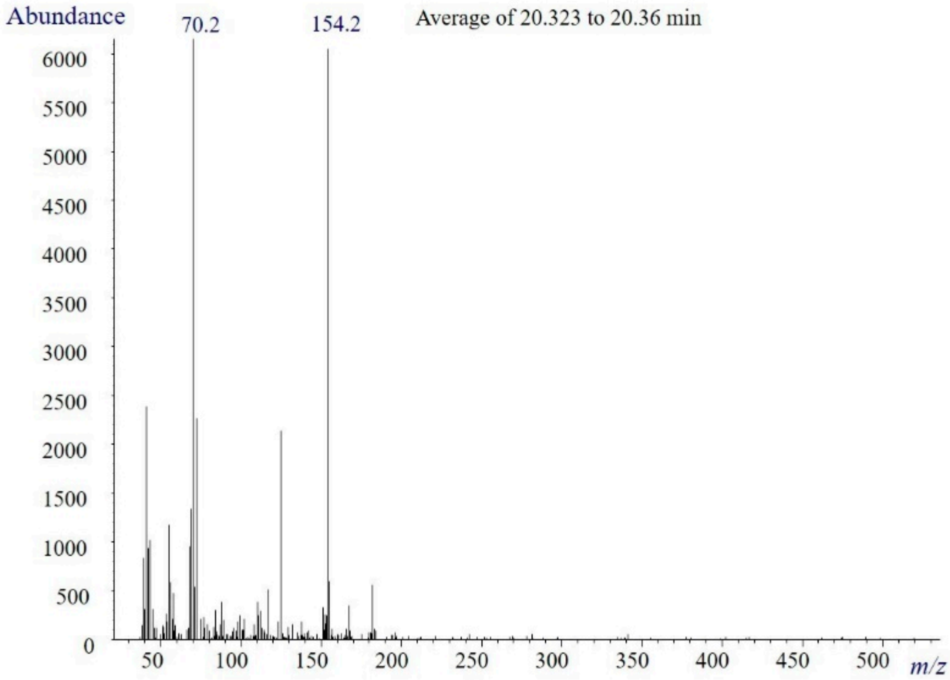

Figure S1e. Mass spectrum of the peak N° 5 (Average of 20.323 to 20.36 min) shown in figure 6 by using gas chromatography-mass spectrometry (GC-MS) technique.

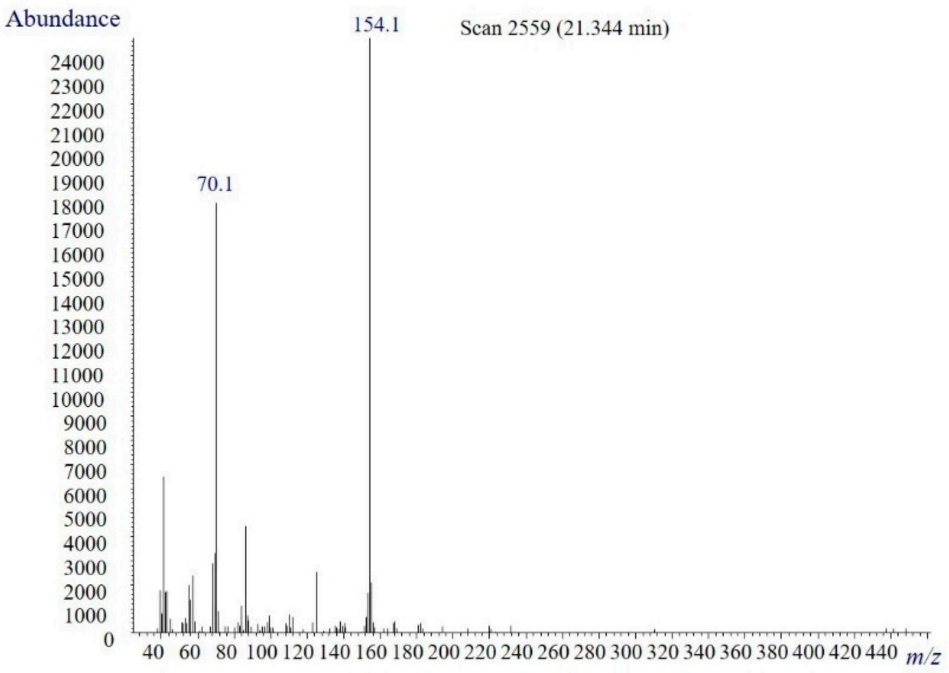

Figure S1f. Mass spectrum of the peak N° 6 (21.344 min) shown in figure 6 by using gas chromatography-mass spectrometry (GC-MS) technique.

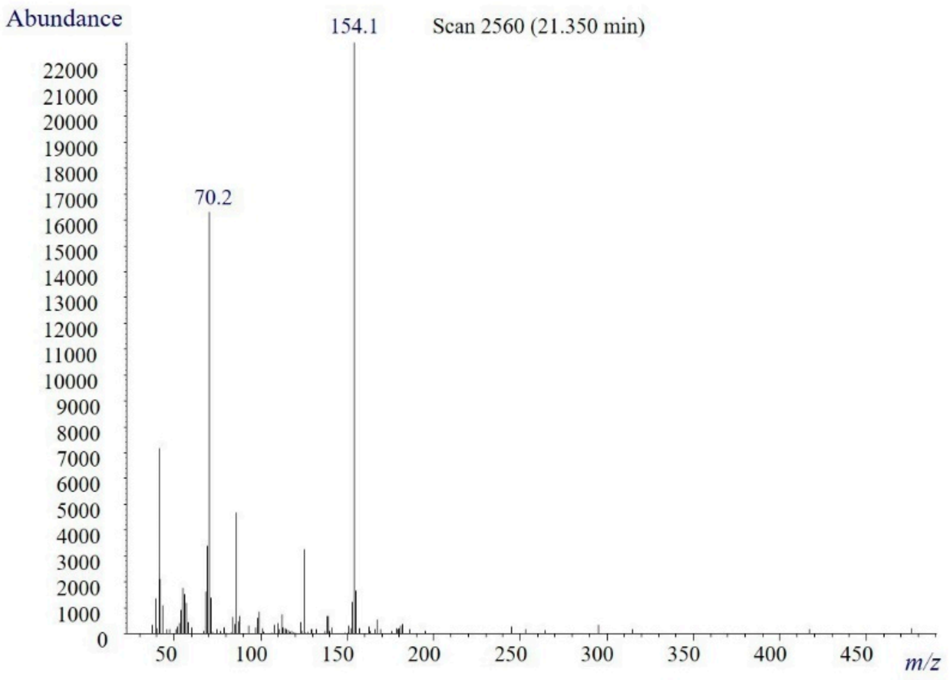

Figure S1g. Mass spectrum of the peak N° 7 (21.35 min) shown in figure 6 by using gas chromatography-mass spectrometry (GC-MS) technique.

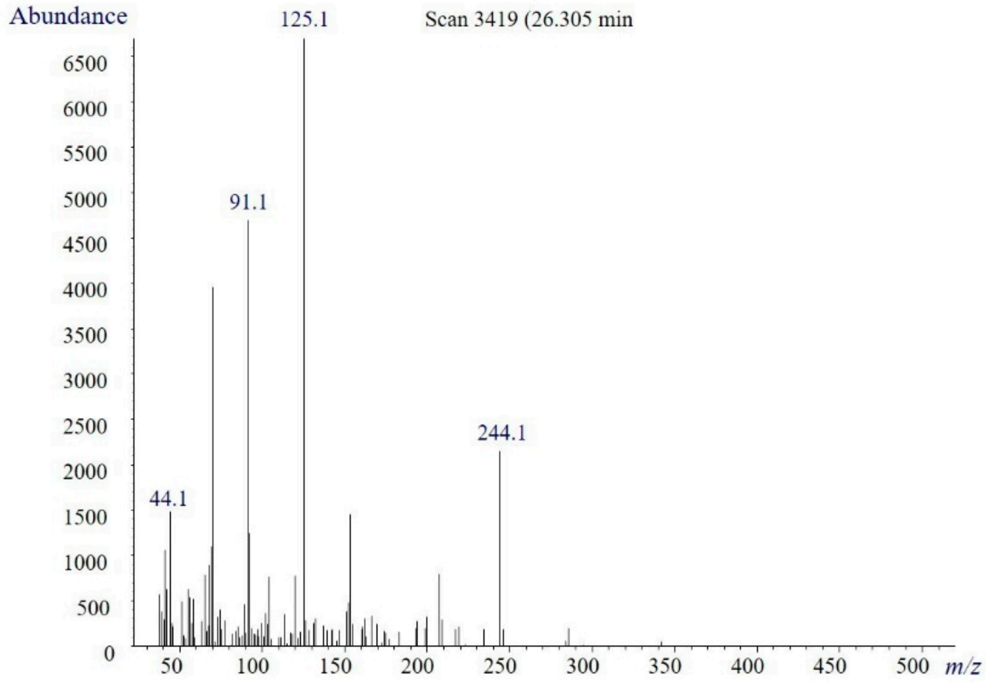

Figure Mass spectrum of the peak N° 8 (26.305 min) shown in figure 6 by using gas chromatography-mass spectrometry (GC-MS) technique.

© 2020 by the authors. Licensee MDPI, Basel, Switzerland. This article is an open access article distributed under the terms and conditions of the Creative Commons Attribution (CC BY) license (http://creativecommons.org/licenses/by/4.0/).
